# Population density mediates induced immune response, but not physiological condition in a well-adapted urban bird

**DOI:** 10.1038/s41598-022-12910-1

**Published:** 2022-06-01

**Authors:** Maciej Kamiński, Amelia Chyb, Piotr Minias

**Affiliations:** Department of Biodiversity Studies and Bioeducation, Faculty of Biology and Environmental Protection, University of Lodz, Banacha 1/3, 90-237 Lodz, Poland

**Keywords:** Urban ecology, Ecophysiology

## Abstract

Thriving under high population density is considered a major feature of urban exploiter species. Nevertheless, population density appears to be a surprisingly overlooked factor in urban ecology studies. High population numbers observed in urban species might promote pathogen transmission and negatively affect health or condition, thus requiring investments in immunocompetence. The feral pigeon *Columba livia domestica* is an example of a successful city-dweller, found in great abundance in large cities across the globe. We investigated the effects of population density on induced immune response (phytohaemagglutinin skin test) and body condition (blood haemoglobin concentration and size-corrected body mass) in 120 feral pigeons, captured along population density gradient in Łódź (central Poland). We found that stronger immune response was associated with higher population density, but was not related to physiological condition and physiological stress (heterophil/lymphocyte ratio). Moreover, condition indices were not associated with population density. However, since pigeon population density was highly correlated with the level of habitat urbanization, we cannot exclude that any density-dependent effects may be mediated by habitat variation. Our results indicate that urban environment, via population density, might exert different selective pressures on immunocompetence and body condition in this successful urban exploiter.

## Introduction

The process of landscape urbanization is generally found to have a detrimental effect on wildlife biodiversity^1,2^, yet increasing numbers of animal species are becoming adapted to urban lifestyles. Living in an urban environment induces changes in morphological, behavioural or ecological traits of wildlife, including birds^3,4^. Urban bird populations must adapt to permanent human presence, distorted photoperiod, noise pollution, non-natural food resources and altered nesting sites. On the other hand, they benefit from alleviated predator pressure, abundant food supplies and milder climate, which can facilitate sedentariness and prolong the breeding period^5–7^. This set of factors often promotes higher population densities in urban areas when compared with natural (non-urban) habitats. In fact, high population density is considered to be one of the most important characteristics of urban species^3,8,9^. Consequently, it seems surprising that the effects of population density on fitness-related traits have rarely been tested in urban birds. Multiple studies on non-urban avian species have reported effects of population density (usually nest density or social group size) on nestling performance^10–13^, but similar studies on adult birds in urban settings are lacking.

Generally, high population densities may be associated with diverse ecological and fitness trade-offs. In large groups, individuals can more efficiently exchange social information, such as locations of food sources^14^. Social groups also facilitate finding a partner and reduce predator threat via increased vigilance and more efficient antipredator reactions^15–17^. On the other hand, high population density can generate fitness-related costs and can pose direct threats to individuals^18^. Living in large social groups causes increased antagonistic intraspecific interactions, elevates social stress and promotes transmission of pathogens, which may lead to deterioration of body condition and reduce fitness^12,19^. While migrant birds are exposed to high population densities only at some part of their annual cycle (either during breeding, migration, or at wintering sites), sedentary urban birds are likely to permanently operate under similar (high) density regimes. Due to the fact that sedentary urban birds can thrive in high local densities, one might expect that they are able to mitigate the negative density-dependent pressures experienced by other species. Alternatively, individuals may suffer from impaired health status under high densities, but instead gain fitness advantage in reproduction. Multiple bird species have been demonstrated to trade their body condition and life span for reproductive success^20–22^, but it seems that this is not the case in urban exploiters, as there is a considerable body of evidence indicating increased longevity in urban birds^3,23–26^.

The feral pigeon *Columba livia domestica* is one of the most well-adapted urban exploiter species and has inhabited cities for centuries. As a “feral” species, its extensive city colonization and distribution across the world were facilitated and preceded by domestication^27^. Artificial selection provided feral pigeons with traits important for colonization of man-made habitats (e.g. reduced shyness toward people^27^). Despite their origin and ongoing introgression from domestic pigeons^28^, the feral pigeon, in most cases, operates in free-living populations under natural selection^29^. Nowadays, feral pigeons are able to dwell in large cities under extreme local densities (often considered to require pest control^30,31^). Due to its abundance, tameness and resilience, this species can be used as a suitable model to study processes of successful urbanization and life-history trade-offs within urban populations (e.g.^32,33^).

**Figure 1:**
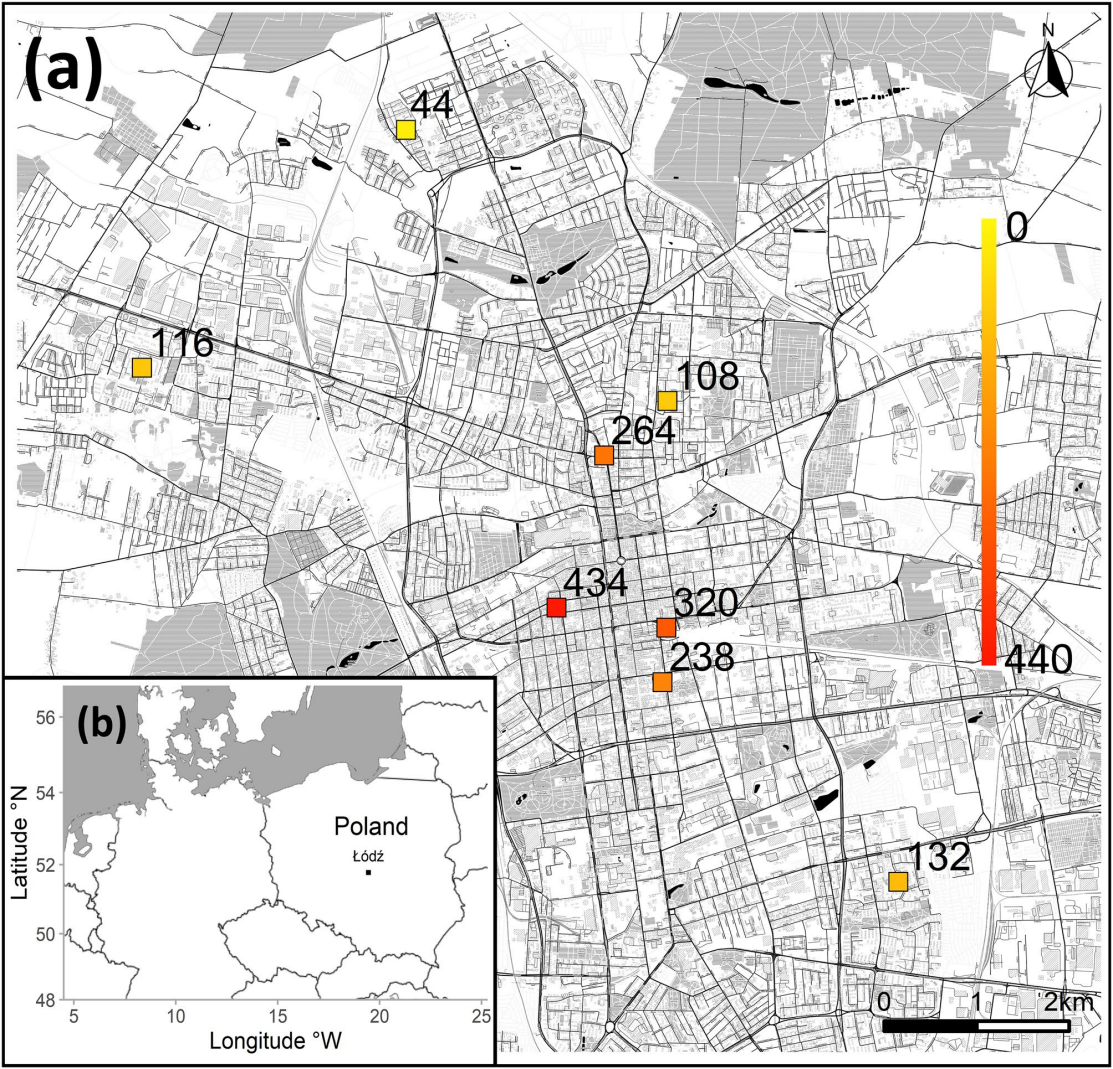
Map of study plots within city of Łódź with numbers of feral pigeons counted at each plot (**a**) and location of Łódź (**b**). The figure was prepared in in R v. 4.0.2 basing on OpenStreetMap tiles (www.openstreetmap.org/copyright, licensed under CC BY-SA).

Here, we aimed to investigate the effects of population density on induced immune response and body condition in feral pigeons. In general, breeding in large densities or large social groups may trigger adaptive investments in immunocompetence, counteracting elevated pathogen transmission^34,35^. Thus, we hypothesized that higher population density (higher number of individuals within study plots) should be associated with stronger induced immune response (measured with phytohaemagglutinin [PHA] skin test), reflecting enhanced immunocompetence. However, induced immune response (henceforth PHA response) may also be affected by physiological stress, because elevated exposure to antagonistic interactions raises the level of corticosteroid hormones and suppresses immune functions^36,37^. If individuals dwelling in larger congregations are subjected to higher physiological stress (measured as heterophil/lymphocyte ratio, henceforth H/L ratio), it may lead to impaired immunocompetence of birds from higher population densities. Finally, the effects of population density on immune response may be mediated by its direct effects on body condition^38,39^. We hypothesized that population density might exert fitness-related costs by decreasing physiological or nutritional condition of individuals, e.g. through stronger competition for food^19,40^, although many sources reported no deteriorated condition of birds living in highly urbanized environments^6,41,42^. To address these hypotheses, we captured 120 individuals of feral pigeon across study plots of varying density (Fig. 1a) and subjected them to the immune response skin test with PHA injection. To obtain two independent condition indices, we measured blood haemoglobin concentration (henceforth HB) and size-corrected body mass (Scaled Mass Index, SMI^43^). The feral pigeon displays several colour morphs, which differ in the extent of melanin plumage coloration and it was demonstrated that PHA response can be stronger in darker feral pigeon morphs^44^. Taking it into account, we controlled for morph variation in our analyses. Research on urban bird ecology often hinges on the habitat urbanization gradient (e.g.^5,45–47^) or on the comparisons between urban and rural populations (e.g.^48–50^). Here, we exclusively focused our research on an urban population, but expected that pigeon density may also be associated with a fine-scale variation in the level of habitat urbanization. Therefore, we also calculated urbanization score to investigate the associations between urban habitat features and our response variables (PHA response, HB and SMI).

## Results

### Induced immune response

**Figure 2:**
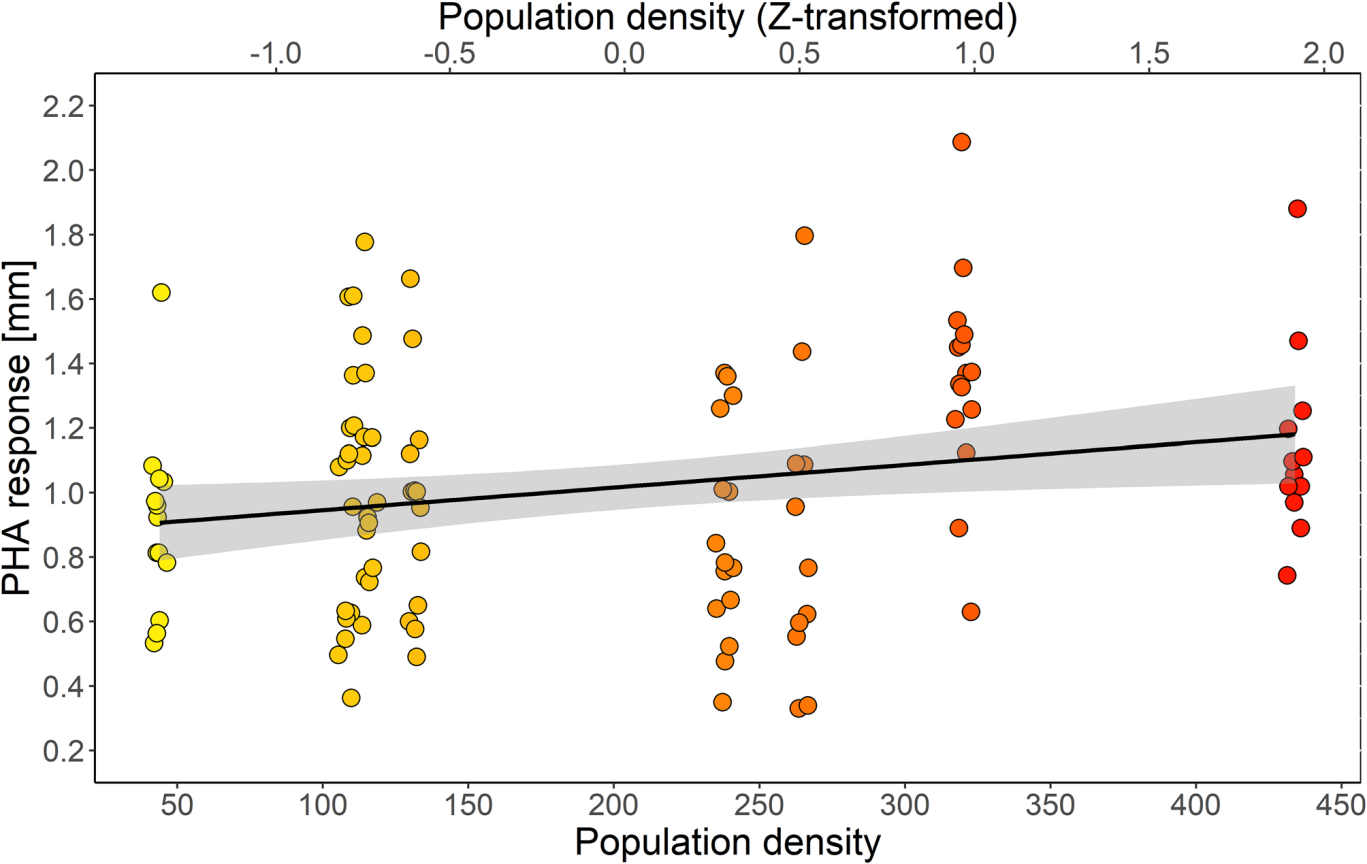
The relationships between induced immune response (PHA response) and feral pigeon population density (number of individuals per plot). The black line and shaded area represents ordinary least squares regression with 95% confidence intervals.

The mean PHA response was 1.02 ± 0.04 [SE], ranging from 0.33 to 2.09 mm. Our analysis indicated that PHA response was positively associated with population density (β = 0.11 ± 0.04, *P* = 0.04, Fig. 2). The effect of wing length was also significant and stronger PHA response was recorded in larger birds (i.e. those with longer wings; β = 0.01 ± 0.006, *P* = 0.04). We found no significant association between PHA response and H/L ratio (*P* = 0.36). Also, all other predictors were not associated with PHA response (all *P* ≥ 0.24), as we did not detect any significant relationships with body condition, plumage darkness score, season, age nor sex (Table 1). Marginal and conditional pseudo-R^2^ values for the model were 0.17 and 0.21, respectively. Urbanization score was marginally non-significant (*P* = 0.069), when used instead of population density in the model (Table S1 in Electronic Supplementary Material).

**Table 1:** The effects of population density, physiological stress (heterophil/lymphocyte ratio, H/L ratio), body size (wing length), plumage darkness score, blood haemoglobin concentration (HB), scaled mass index (SMI), season, sex and age on induced (PHA) immune response, as assessed with generalized linear mixed model fitted with Restricted Maximum Likelihood (REML).

Predictor	β ± SE	t	*P*	*R*_*m*_^*2*^
Intercept	− 2.56 ± 1.53	− 1.67	0.097	–
**Population density (Z-transformed)**	**0.11 ± 0.04**	**2.58**	**0.043**	**0.086**
H/L ratio (Z-transformed)	− 0.03 ± 0.04	− 0.92	0.359	0.008
**Wing length**	**0.01 ± 0.01**	**2.13**	**0.036**	**0.041**
Plumage darkness score	0.02 ± 0.03	0.44	0.655	0.002
HB	0.002 ± 0.002	0.90	0.370	0.007
SMI	0.001 ± 0.001	1.19	0.238	0.013
Season (summer vs. winter)	0.05 ± 0.11	0.43	0.667	0.002
Sex (female vs. male)	− 0.05 ± 0.08	− 0.66	0.508	0.004
Age (immature vs. adult)	− 0.07 ± 0.12	− 0.60	0.548	0.003

The plot identity was used as the random factor. The semi-partial *R*_*m*_^*2*^ represents the percentage of variance explained by each predictor. Significant predictors are marked in bold.

### Condition indices

**Figure 3:**
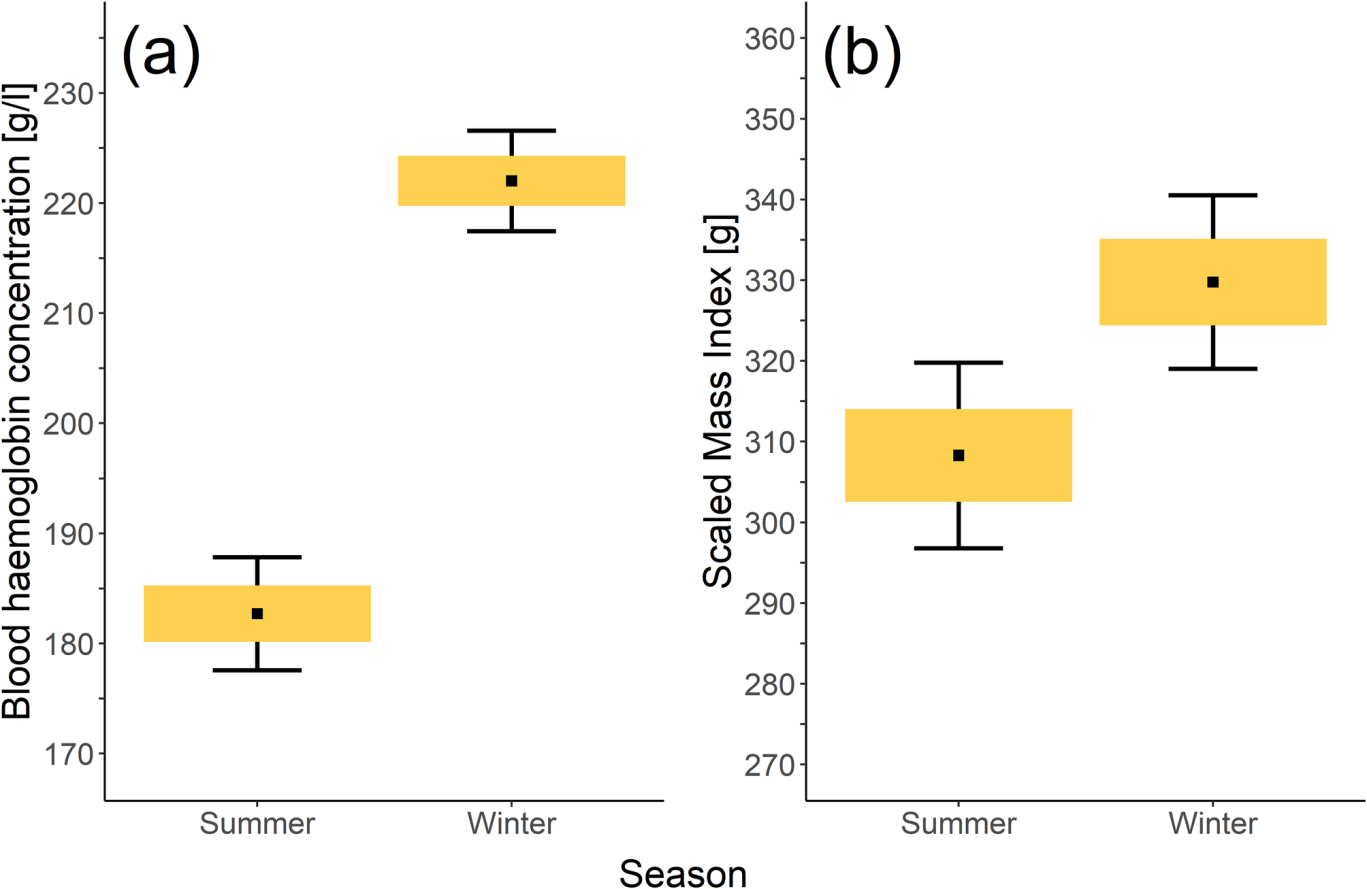
Comparison of (**a**) blood haemoglobin concentration and (**b**) Scaled Mass Index of feral pigeons in summer and winter. Central points indicate mean value, boxes and whiskers represent standard error and 95% confidence intervals, respectively.

In winter, feral pigeons had significantly higher HB levels when compared to the summer period (β = 36.76 ± 3.19, *P* < 0.001, Fig. 3). In contrast to PHA response, HB was not associated with population density (*P* = 0.60) or with H/L ratio (*P* = 0.16). The effect of age was significant, with adults having higher HB than immature birds (Table 2). We found no evidence for relationships of HB with plumage darkness (*P* = 0.36) or sex (*P* = 0.25). Marginal and conditional pseudo-R^2^ values for the model were estimated at 0.64, as the random effect (study plot) explained zero variance. The model with urbanization score replacing population density yielded similar results (Table S2 in Electronic Supplementary Material).

**Table 2:** The effects of population density, physiological stress (heterophil/lymphocyte ratio, H/L ratio), body size (wing length), plumage darkness score, season, sex and age on blood haemoglobin concentration, as assessed with generalized linear mixed model fitted with Restricted Maximum Likelihood (REML). The plot identity was used as the random factor.

Explanatory variable	β ± SE	t	*P*	*R*_*m*_^*2*^
**Intercept**	**115.36 ± 43.00**	**2.68**	**0.009**	**–**
Population density (Z-transformed)	− 0.84 ± 1.63	− 0.52	0.606	0.002
H/L ratio (Z-transformed)	− 2.30 ± 1.64	− 1.40	0.164	0.018
Wing length	0.24 ± 0.19	1.25	0.213	0.014
Plumage darkness score	− 1.47 ± 1.61	− 0.91	0.363	0.008
**Season (summer vs. winter)**	**36.76 ± 3.19**	**11.52**	**< 0.001**	**0.554**
Sex (female vs. male)	3.76 ± 3.27	1.15	0.254	0.012
**Age (immature vs. adult)**	**17.04 ± 4.59**	**3.72**	**< 0.001**	**0.114**

The semi-partial *R*_*m*_^*2*^ represents the percentage of variance explained by each predictor. Significant predictors are marked in bold.

The SMI was also not associated with population density (*P* = 0.75). Similar to HB, SMI values were higher in the winter than summer season (β = 19.09 ± 7.56, *P* = 0.01, Fig. 3). Also, males and adults had significantly higher SMI (Table 3). We found no association between SMI and H/L ratio (*P* = 0.37) or plumage darkness (*P* = 0.56). Marginal and conditional pseudo-R^2^ values were 0.20 (variance explained by the random effect was close to zero). Replacing population density with urbanization score produced similar results in terms of predictor significance and their effect sizes (Table S3 in Electronic Supplementary Material).

**Table 3:** The effects of population density, physiological stress (heterophil/lymphocyte ratio, H/L ratio), plumage darkness score, season, sex and age on scaled mass index, as assessed with generalized linear mixed model fitted with Restricted Maximum Likelihood (REML).

Explanatory variable	β ± SE	t	*P*	*R*_*m*_^*2*^
**Intercept**	**273.39 ± 12.37**	**22.10**	**< 0.001**	**–**
Population density (Z-transformed)	− 1.26 ± 3.86	− 0.33	0.745	0.001
H/L ratio (Z-transformed)	− 3.52 ± 3.86	− 0.91	0.367	0.008
Plumage darkness score	2.25 ± 3.81	0.59	0.556	0.003
**Season (summer vs. winter)**	**19.09 ± 7.55**	**2.53**	**0.013**	**0.056**
**Sex (female vs. male)**	**20.35 ± 7.56**	**2.69**	**0.008**	**0.064**
**Age (immature vs. adult)**	**24.87 ± 10.36**	**2.40**	**0.018**	**0.051**

The plot identity was used as the random factor. The semi-partial *R*_*m*_^*2*^ represents the percentage of variance explained by each predictor. Significant predictors are marked in bold.

## Discussion

Our results provide evidence for a positive relationship between population density and cell-mediated immunocompetence in the feral pigeon, a successful city dweller. In contrast, we found no support for the hypothesis that physiological or nutritional condition is shaped by population numbers. Also, neither body condition nor physiological stress (H/L ratios) covaried with induced immune response. This suggests that density-dependent patterns in immunocompetence of feral pigeons are not primarily driven by variation in physiological or nutritional condition. We also found that larger birds (i.e. with greater wing length) developed stronger swelling in the PHA injection spot. This pattern was previously observed at the interspecific level in birds^51^ and it might be related to the volume of tissue exposed to PHA, however physiological mechanism of this association remains unclear^51^. Additionally, since living in high population density is a characteristic feature of many urban populations, it is often difficult to disentangle the effects of population density and habitat urbanization. In this study, the population density and urbanization score were highly correlated and we could not effectively determine whether and how the observed density-dependent patterns were mediated by the background variation in habitat urbanization.

Dwelling in large groups of conspecifics imposes higher risk of disease^52,53^. For instance, bird feeders and dense flocks of birds around them enhance horizontal pathogen transmission^54,55^. This might promote elevated resource investment in immunocompetence to counteract higher pathogen pressure. Urban birds have larger bursa of Fabricius than non-urban species^56^, which contributes to enhanced B cell-mediated immunity. Audet et al.^46^ also showed stronger PHA response in urban Barbados bullfinches *Loxigilla barbadensis* compared to rural birds, and urban great tits *Parus major* exhibited significantly higher expression of genes related to adaptive and innate immunity^57^. Our results, demonstrating that population numbers are positively associated with PHA response, are congruent with these findings, although it needs to be acknowledged that we sampled birds exclusively at urban study plots, despite some fine-scale variation in urbanization degree. Furthermore, body condition indices were not related with population density and PHA response per se was not mediated by physiological (HB) or nutritional (SMI) condition. Both indices, to some extent, should reflect the quantity and quality of food resources available for individuals. It was demonstrated that energetic and nutritional costs of mounting an immune response are rather low^58^. Consequently, we may conclude that these two fitness-related traits of feral pigeons (immunocompetence and condition) are independently shaped by urban environment. In fact, the level of habitat urbanization can also impose a direct effect on immunity or condition. The prevalence of avian viruses and bacteria—but not helminths and ectoparasites—increases along urbanization gradient^25^. Sepp et al.^25^ also hypothesized that urban birds have longer lifespan and invest more in self-maintenance e.g. by increased immunocompetence. Our results are in accordance with these predictions and, additionally, they suggest that population density might be involved in mediating the influence of habitat urbanization on immune traits.

One should notice that the population density, although statistically significant, did not impose a strong effect on PHA response. Although the population density accounted for about 8% of explained variance in PHA response (Table 1), the variability within study plots was high (Fig. 2). Therefore, it seems that induced cellular immune response is also influenced by other factors, not included in our analyses. We also did not quantify the prevalence of pathogens in our population. It was suggested^59^ that parasite infestation could influence indices of immunocompetence (e.g. PHA response). Although Biard et al.^59^ did not found any general effect of parasites on PHA response, there were some interacting effects with infestation degree. Also, Leclaire et al.^60^ showed that bacterial load on feathers (but not haemosporidians) was associated with higher PHA response. We acknowledge that information on the variation in pathogen prevalence and their transmission rates may provide further insights into density-dependent patterns of pigeon immunocompetence. Other factors expected to explain variation in avian PHA response, like immunosenescence, social hierarchy or gut microbiota^61^, were also not included in our study.

The H/L ratio is an honest indicator of physiological stress^62^. Competition and other antagonistic interactions with conspecifics may be considered major stressors^63,64^. Nevertheless, many other stress factors can simultaneously contribute to elevated H/L ratios: e.g. infections^65^, malnutrition^66^ or the level of habitat urbanization^67^. An extensive exposure to stress, irrespective of its sources, has a negative effect on immunity^68,69^, since it suppresses inflammatory response and diminishes the number of T-cells in circulating blood. Therefore, it seems reasonable to expect a negative association between PHA response and H/L ratios, as reported for several avian species^36,70,71^, albeit we did not detect any significant association of this kind in the pigeon. In the European starling *Sturnus vulgaris* (also an urban exploiter species) individuals under experimentally evoked chronic psychological stress did not suppress PHA response in comparison to unstressed birds^72^. At the same time, H/L ratio of our study pigeons was negatively associated with another haematic parameter (HB concentration), but not with size-corrected body mass (SMI), indicating that some components of body condition may be more affected by stress than the others. Supposedly, gregarious city exploiters like feral pigeon could adaptively down-regulate the negative physiological consequences of persistent exposure to stressors and several studies reported diminished physiological stress response of urban birds^5,48,73,74^, but see also^75^.

Differences between females and males in PHA response may occur in different stages of the annual cycle, either during breeding or non-breeding period^76^. We did not have information on the breeding status of our sampled pigeons, but the species is considered to breed almost year-round, with a reproductive peak in spring and summer^77,78^. We did not find any significant differences in PHA response between summer and winter, or between sexes and age classes, yet physiological (HB) and nutritional (SMI) condition was higher in winter than summer (Fig. 3). It was postulated that birds exposed to cold temperatures during winter season should elevate HB to intensify oxygen transportation required for increased thermoregulation^79^, but see also^80^.

Finally, the expression of melanin-based coloration is expected to covary with many fitness-related traits, including immunocompetence^81^. Studying pleiotropic associations between melanin pigmentation and immune functions, Jacquin et al.^44^ reported that intensity of melanin coloration among feral pigeon morphs was related with PHA response and parasite infestation. However, our analysis did not reveal significant associations of melanin-based coloration score with PHA response. Neither did we observe any associations between morphs and physiological condition.

In summary, we provided correlational evidence that population density might modulate induced cellular immune response in the feral pigeon, as stronger immune response was mounted by individuals living in higher population density. At the same time, our study did not find evidence for density-dependent variation in body condition. All this suggests, that feral pigeons are able to mitigate physiological costs of group living and high densities. The pigeon population density was highly correlated with level of landscape urbanization. Thus, using multivariate modelling, we were unable to effectively separate the effects of population density and urbanization on PHA response, which would require an experimental study design. Studies investigating density-dependence in fitness components and fitness-related traits of urban birds clearly deserve further attention under broader phylogenetic framework, and should particularly focus on an effective separation between the effects of population density and urban habitat features. We recommend that population density should be considered as a relevant factor in urban ecology studies, especially of urban exploiters.

## Methods

### Population density estimation

The experiment was conducted in the population of feral pigeons from Łódź (central Poland, 51° 46′ 36″ N 19° 27′ 17″ E, Fig. 1b). This is a former industrial city with a population of 672,100 inhabitants^82^, which rapidly developed in XIX century. Before bird sampling, we assessed feral pigeon population densities on eight plots of 300 × 300 m dimensions. Four plots were located in central area of the city and they represented locations with roughly the highest feral pigeon densities (personal obs.). The dominant type of development at these plots included late 19th and early 20th century four-floor tenements. The other four plots were located in districts of four-floor housing blocks built in 1970s and 1980s outside the city centre. On each plot (both in the city centre and in residential areas) there were potential breeding, roosting and feeding sites for pigeons and birds were regularly provided with anthropogenic food by local inhabitants. The population density of feral pigeons on the plots was surveyed at the turn of February 2020, prior to the peak of breeding season. During the surveys, we aimed to count all feral pigeon individuals present within plot borders. All plot surveys were carried out by one author (MK) to increase repeatability. The plots were inspected during favourable weather and each visit lasted ca. 45 min. Overflying birds were not included in the counts and very large flocks were counted from photographs taken on the spot. Overall, pigeon numbers ranged from 44 to 434 individuals per plot (Fig. 1a), with over three-fold difference in the mean numbers between city centre (314 ± 19.46 [SE] individuals) and housing block districts (100 ± 8.64 [SE] individuals). The number of individuals counted on each plot was used as an index of population density. Since pigeons are highly sedentary^83^, we presumed that our count results should reflect relatively stable numbers of pigeons occupying particular roosting and feeding sites (plots).

### Field procedures

The study was conducted in compliance with the ARRIVE guidelines. It was performed in accordance with the national legislation and was approved by the Local Bioethical Commission for Experiments on Animals in Łódź (1/ŁB154/2020) and the General Directorate for Environmental Protection in Poland (DZP-WG.6401.15.2020.KS). Birds were sampled in two distinct periods: summer (June to August 2020, 7 individuals per plot: N = 56) and winter (late November 2020 to early March 2021, 8 individuals per plot: N = 64). In both seasons we captured individuals on the same eight plots. We used wheat grain, dry pea and corn as a bait. Pigeons were captured in hands, if they were tame enough, or with fishing line noose trap. The noose trap is fairly common in catching waterbirds^84,85^ and it proved to be efficient and safe for feral pigeons. To avoid recaptures, caught birds were individually marked with plastic bands used for domestic pigeons. We only captured four most common pigeon plumage morphs^86^: Blue-bar (N = 30), Blue-checker (N = 42), Blue T-pattern (N = 33) and Spread Black (N = 15). The morphs were ranked according to plumage darkness score^44^. Individuals from other morphs and with irregular plumage phenotypes (e.g. with patches of white feathers) were excluded from study procedures and were immediately released, if captured in noose trap. Age was determined as immature (N = 20) or adult (N = 100). Birds were classified as immature by the presence of at least two of the following traits: pinkish bill with underdeveloped pinkish operculum, dark scales on legs, lack of iridescence on neck feathers, brownish or pale orange iris and brownish or buff edges on the lesser coverts. We gathered basic biometric measurements including wing length (measured to the nearest 1 mm with a ruler), head, bill and tarsus (measured to the nearest 0.1 mm with a calliper) and body mass (measured on electronic scale to the nearest 1 g).

Blood samples were collected by puncturing the ulnar vein with 0.6 mm gauge needle. We collected ca. 40 µl of blood into Eppendorf tube filled with 96% ethanol for molecular sexing. A droplet of blood was used to prepare a blood smear on a microscope slide and ca. 5 µl were collected into a disposable Hemocue microcuvette for immediate HB measurement in a portable photometer HemoCue Hb 201 + (HemoCue, Ängeholm, Sweden). This device relies on azide-methaemoglobin method and is suitable for measuring blood haemoglobin concentration in wild birds^87^. HB is considered a robust indicator of physiological condition^88^. After sampling, pigeons were individually placed in pet carriers and transported for PHA challenge (within 6 h after capturing).

### PHA challenge

Immediately after transportation to the facilities (University of Łódź), we performed PHA response skin test on captured birds. The PHA is a lectin and it has mitogenic properties. PHA injection is extensively used to evaluate immunocompetence in wild animals by obtaining measurement of induced cellular immune response^51,89–91^. It is commonly regarded as a measure of T-cell response, since PHA triggers mitosis in T-cells, but PHA-induced immune reaction also involves other types of white blood cells^91–93^. To perform measurements of PHA-induced immune response we first removed down feathers from left wing patagium and measured its thickness three times to the nearest 0.01 mm using pressure-sensitive micrometer (Mitutoyo, Kawasaki, Japan). Next, we injected left wing patagium with 0.2 mg PHA (Phytohaemagglutinin PHA-P L8754, Sigma-Aldrich Co., St. Louis, MO, USA) dissolved in 0.05 ml of phosphate-buffered saline (PBS; Sigma-Aldrich Co.). For injections we used disposable syringes with 0.33 mm gauge needle. All birds were individually kept in pet carriers (48 × 31.5 × 33 cm) in a dark room (20–22 °C), without human presence. After 24 h, we checked the increase of patagium web thickness, repeating the measurement three times. The difference between mean patagium thickness after and before PHA injection was used as an index of induced immune response. Instantly after the final measurement, birds were set free and offered with optional feeding. None individual showed any symptoms of condition deterioration after treatment. Post-treatment observations indicated that most pigeons returned to their original plots.

### Molecular sexing

To determine the sex of sampled individuals, we relied on molecular sexing method^94^. DNA was isolated with Bio-Trace DNA Purification Kit (EURx, Gdańsk, Poland, according to the kit protocol). We successfully used P2/P8 primers designed for CHD-based sex identification in non-ratite birds^95^. The products of PCR were separated by 2% agarose gel electrophoresis, allowing to recognize females with two bands and males with one band. The sex ratio within our sample did not deviate from parity (N females = 59, N males = 61, G = 0.033, *P* = 0.86).

### Leukocyte profiles

The blood smears were stained with HEMAVET kit (Kolchem, Łódź, Poland), which uses modified Romanowsky staining technique. All blood smears were surveyed under a light microscope (×1000 magnification with oil immersion) to evaluate leukocyte profiles, i.e. a random sample of 100 white blood cells from a smear was differentiated to identify five types of leukocytes: lymphocytes, heterophils, eosinophils, basophils, and monocytes. The proportion of heterophil to lymphocyte number (H/L ratio) was used as a proxy of physiological stress. For technical reasons (faulty smearing or drying) we failed to assess leukocyte profiles in 11 individuals. All blood smears were surveyed by one author (AC). To quantify repeatability, 25 randomly chosen blood smears were assessed twice, yielding highly repeatable measurements (*r* = 0.84, *P* < 0.001). One individual with very high H/L ratio (> 6 SD) was considered an outlier and removed from further analyses.

### Scaled mass index

Based on biometric measurements, we calculated Scaled Mass Index (SMI) to obtain a size-corrected index of body condition that is expected to reflect nutritional state and fat reserves. The SMI was calculated according to the equation developed by Peig and Green^43^. Wing length was chosen as a linear body measurement, since it had the highest ln-ln correlation with body mass (*r* = 0.68). The arithmetic mean of wing length was used as L_0_ constant. The formula was developed separately for females and males.

### Urbanization score

To account for differences in urban habitat structure between study plots, we collected data on three urbanization features: total unpaved green area, total building area (both to the nearest 1 m^2^) and total number of trees. The area measurements were performed in QGIS 3.22.2 (QGIS Geographic Information System, QGIS Association). The information on tree number was derived from Regional Tree Crown Map (MGGP Aero, Tarnów, Polska). This online map, created with LiDAR technology, contains information on trees which meet the minimum height parameter of 4 m and the minimum canopy area of 9 m^2^. The data on green area, building area and tree number were combined into a univariate measure with principal component analysis (PCA). The urbanization score was expressed as the mean-centered first PCA component (PC1), which explained 82% of total variance in the data and was the only one meeting Kaiser’s criterion^96^. Higher PC1 values were associated with increased proportion of buildings, lower tree number and decreased green area cover.

### Statistical analyses

Data analyses were performed in R v. 4.0.2 (R Foundation for Statistical Computing Vienna, Austria). The dataset consisted of 108 individuals with a complete set of measurements (N_summer_ = 56, N_winter_ = 52). To investigate predictors of PHA response and body condition we used general linear mixed models, as fitted with restricted maximum likelihood (REML) using *lmer* function from the *lme4* package^97^. The *lmerTest* package was used to infer predictor *P* values^98^. The response variables (PHA response, HB, and SMI) were normally distributed. The number of birds on plots (density) and H/L ratio were not normally distributed and they were Z-standardized. We detected no multicollinearity among independent variables (|*r*|< 0.33), except urbanization score and population density (r = 0.96). As a consequence, including both population density and urbanization score in the same model led to severe multicollinearity, indicated by high values of Variance Inflation Factor (VIF > 12.7). Thus, we ran separate models with either population density or urbanization score included as a covariate. In the final PHA response models we also included HB and SMI condition indices, H/L ratio, wing length (to account for variation in body size), and plumage darkness score (ranked basing on plumage morphs^44^) as covariates. Season (two levels: summer and winter), sex and age were included as fixed factors, while plot identity was used as a random factor. To test for associations of population density/urbanization with body condition, we run models with HB and SMI as response variables. In both models we used population density/urbanization score, H/L ratio, plumage darkness score (covariates), season, sex and age (fixed factors) and plot identity (random factor). In HB model we also added wing length as a proxy of body size (not included in the SMI model, as variation in body size was removed by SMI estimates). We used *r2beta* function from *r2glmm* package^99^ to obtain semi-partial R^2^ statistics for each fixed predictor in the models. The R^2^ was calculated applying Nakagawa and Schielzeth approach^100^. We used MuMIn package^101^ to infer marginal and conditional pseudo-R^2^ values for the models, which inform on the percentage of response variable variance explained by fixed effects and both fixed and random effects, respectively. Figures were prepared with *ggplot2* and *ggmaps* packages^102,103^.

## Supplementary Information

Supplementary Information 1.

Supplementary Information 2.

Supplementary Information 3.

## Data Availability

Raw data and R code are available as Electronic Supplementary Material.
