# Supplementary material for: Population density mediates induced immune response, but not physiological condition in a well-adapted urban bird
Source: Sci Rep. 2022 Jun 1;12:9150. doi: 10.1038/s41598-022-12910-1 (PMC9160275; doi:10.1038/s41598-022-12910-1)
Supplement: Supplementary file 1 — Supplementary Information 1. [file 41598_2022_12910_MOESM1_ESM.docx]

Table S1. The effect of urbanization score, physiological stress (heterophils/leukocytes ratio, H/L ratio), body size (wing length), plumage darkness score, blood haemoglobin concentration (HB), scaled mass index (SMI), season, sex and age on induced immune response, as generalized linear mixed model fitted with Restricted Maximum Likelihood (REML). The plot identity was used as the random factor. The semi-partial *R_m_^2^* represents the percentage of variance explained by each predictor. Significant predictors are marked in bold. The marginal R^2^ (variance explained only by fixed effects) and conditional R^2^ (total variance explained altogether by fixed and random effects) were 0.16 and 0.21, respectively.

| Predictor | β ± SE | t | *P* | *R_m_^2^* |
| --- | --- | --- | --- | --- |
| Intercept | -2.46 ± 1.54 | -1.60 | 0.112 | - |
| Urbanization score | 0.07 ± 0.03 | 5.88 | 0.069 | 0.074 |
| H/L ratio (Z-transformed) | -0.027 ± 0.04 | -0.76 | 0.449 | 0.005 |
| **Wing length** | **0.01 ± 0.01** | **2.05** | **0.043** | **0.038** |
| Plumage darkness score | 0.01 ± 0.03 | 0.42 | 0.674 | 0.002 |
| HB | 0.002 ± 0.002 | 0.92 | 0.362 | 0.007 |
| SMI | 0.001 ± 0.001 | 1.18 | 0.239 | 0.013 |
| Season (summer vs. winter) | 0.05 ± 0.11 | 0.44 | 0.663 | 0.002 |
| Sex (female vs. male) | -0.05 ± 0.08 | -0.69 | 0.556 | 0.003 |
| Age (immature vs. adult) | -0.08 ± 0.12 | -0.68 | 0.495 | 0.004 |

Table S2. The effect of urbanization score, physiological stress (heterophils/leukocytes ratio, H/L ratio), body size (wing length), plumage darkness score, season, sex and age on blood haemoglobin concentration, as generalized linear mixed model fitted with Restricted Maximum Likelihood (REML). The plot identity was used as the random factor. The semi-partial *R_m_^2^* represents the percentage of variance explained by each predictor. Significant predictors are marked in bold. Both marginal R^2^ (variance explained only by fixed effects) and conditional R^2^ (total variance explained altogether by fixed and random effects) were 0.64.

| Explanatory variable | β ± SE | t | *P* | *R_m_^2^* |
| --- | --- | --- | --- | --- |
| **Intercept** | **115.38 ± 42.85** | **2.69** | **0.008** | **-** |
| Urbanization score | -0.75 ± 1.03 | -0.73 | 0.469 | 0.005 |
| H/L ratio (Z-transformed) | -2.29 ± 1.62 | -1.41 | 0.161 | 0.018 |
| Wing length | 0.24 ± 0.19 | 1.26 | 0.212 | 0.015 |
| Plumage darkness score | -1.45 ± 1.60 | -0.90 | 0.368 | 0.008 |
| **Season (summer vs. winter)** | **36.74 ± 3.19** | **11.53** | **<0.001** | **0.554** |
| Sex (female vs. male) | 3.73 ± 3.27 | 1.14 | 0.256 | 0.012 |
| **Age (immature vs. adult)** | **17.03 ± 4.57** | **3.73** | **<0.001** | **0.115** |

Table S3. The effect of urbanization score, physiological stress (heterophils/leukocytes ratio, H/L ratio), plumage darkness score, season, sex and age on scaled mass index, as generalized linear mixed model fitted with Restricted Maximum Likelihood (REML). The plot identity was used as the random factor. The semi-partial *R_m_^2^* represents the percentage of variance explained by each predictor. Significant predictors are marked in bold. Both marginal R^2^ (variance explained only by fixed effects) and conditional R^2^ (total variance explained altogether by fixed and random effects) were 0.20.

| Explanatory variable | β ± SE | t | *P* | *R_m_^2^* |
| --- | --- | --- | --- | --- |
| **Intercept** | **273.49 ± 12.37** | **22.22** | **<0.001** | **-** |
| Urbanization score | -1.54 ± 2.43 | -0.64 | 0.527 | 0.004 |
| H/L ratio (Z-transformed) | -3.40 ± 3.84 | -0.89 | 0.378 | 0.007 |
| Plumage darkness score | 2.27 ± 3.81 | 0.60 | 0.551 | 0.003 |
| **Season (summer vs. winter)** | **19.04 ± 7.54** | **2.32** | **0.013** | **0.056** |
| **Sex (female vs. male)** | **20.27 ± 7.54** | **2.69** | **0.008** | **0.063** |
| **Age (immature vs. adult)** | **24.66 ± 10.31** | **2.39** | **0.019** | **0.051** |


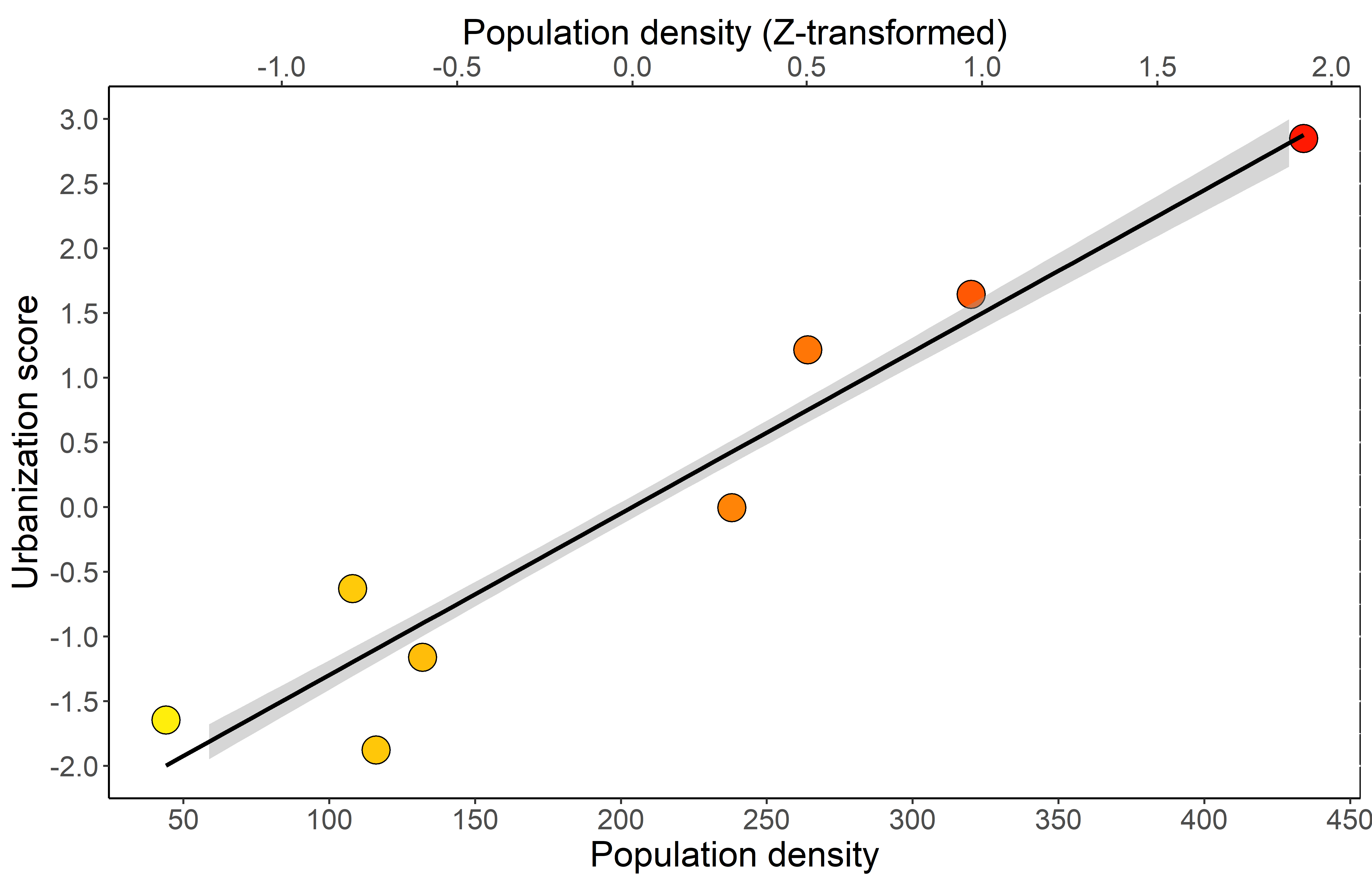


Figure S1. The relationships between urbanization score and feral pigeon population density (number of individuals per plot). The black line and shaded area represents ordinary least squares regression with 95% confidence intervals.
